# (Diethyl­enetriamine)bis­(theophyllinato)zinc(II) dihydrate

**DOI:** 10.1107/S1600536809015013

**Published:** 2009-04-30

**Authors:** Béla Mihály, Edit Forizs, Attila-Zsolt Kun, Ioan Silaghi-Dumitrescu

**Affiliations:** a Faculty of Chemistry and Chemical Engineering, Babes-Bolyai University, 11 Arany János Street, RO-400028 Cluj-Napoca, Romania

## Abstract

In the title compound, [Zn(C_7_H_7_N_4_O_2_)_2_(C_4_H_13_N_3_)]·2H_2_O, the Zn^II^ ion is penta­coordinated by three N atoms of the diethyl­enetriamine ligand and one N atom of each of the two theophyllinate anions in a distorted trigonal-bipyramidal geometry. The Zn—N distances range from 2.076 (3) to 2.221 (3) Å. The crystal packing is stabilized by O—H⋯O, O—H⋯N and N—H⋯O hydrogen bonds involving the theophylline and diethyl­enetriamine ligands and uncoordinated water mol­ecules.

## Related literature

For the isostructural copper(II) compound, see Sorrell *et al.* (1976 ▶). For the theophylline mol­ecule acting as a monodentate anionic ligand, see: Begum & Manohar (1994 ▶); Birdsall & Zitzman (1979 ▶); Bombicz *et al.* (1997 ▶).
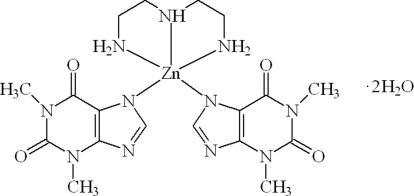


## Experimental

### 

#### Crystal data

[Zn(C_7_H_7_N_4_O_2_)_2_(C_4_H_13_N_3_)]·2H_2_O
*M* *_r_* = 562.91
Monoclinic, 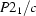

*a* = 18.4655 (13) Å
*b* = 8.2603 (6) Å
*c* = 15.9252 (12) Å
β = 98.904 (1)°
*V* = 2399.8 (3) Å^3^
*Z* = 4
Mo *K*α radiation
μ = 1.08 mm^−1^
*T* = 297 K
0.26 × 0.25 × 0.15 mm

#### Data collection

Bruker SMART APEX diffractometer
Absorption correction: multi-scan (*SADABS*; Bruker, 2001 ▶) *T*_min_ = 0.766, *T*_max_ = 0.854
16801 measured reflections
4214 independent reflections
3800 reflections with *I* > 2σ(*I*)
*R*_int_ = 0.041

#### Refinement

*R*[*F* ^2^ > 2σ(*F* ^2^)] = 0.049
*wR*(*F* ^2^) = 0.115
*S* = 1.15
4214 reflections
349 parameters
5 restraints
H atoms treated by a mixture of independent and constrained refinement
Δρ_max_ = 0.53 e Å^−3^
Δρ_min_ = −0.34 e Å^−3^

### 

Data collection: *SMART* (Bruker, 2007 ▶); cell refinement: *SAINT-Plus* (Bruker, 2007 ▶); data reduction: *SAINT-Plus*; program(s) used to solve structure: *SHELXS97* (Sheldrick, 2008 ▶); program(s) used to refine structure: *SHELXL97* (Sheldrick, 2008 ▶); molecular graphics: *DIAMOND* (Brandenburg & Putz, 1999 ▶); software used to prepare material for publication: *publCIF* (Westrip, 2009 ▶).

## Supplementary Material

Crystal structure: contains datablocks I, global. DOI: 10.1107/S1600536809015013/fi2075sup1.cif

Structure factors: contains datablocks I. DOI: 10.1107/S1600536809015013/fi2075Isup2.hkl

Additional supplementary materials:  crystallographic information; 3D view; checkCIF report

## Figures and Tables

**Table 1:** Hydrogen-bond geometry (Å, °)

*D*—H⋯*A*	*D*—H	H⋯*A*	*D*⋯*A*	*D*—H⋯*A*
O5—H1⋯N8^i^	0.83 (2)	2.01 (3)	2.835 (5)	177 (3)
O5—H2⋯O4	0.82 (6)	2.00 (6)	2.801 (5)	167 (6)
O6—H3⋯O2	0.82 (2)	2.10 (4)	2.914 (4)	169 (4)
O6—H4⋯N4^ii^	0.81 (5)	2.16 (6)	2.957 (5)	168 (4)
N9—H9*A*⋯O6	0.90	2.23	3.096 (5)	161
N9—H9*B*⋯O3^iii^	0.90	2.42	3.213 (4)	147 (2)
N11—H11*B*⋯O3^iv^	0.90	2.07	2.962 (4)	169 (2)
N10—H10⋯O1^v^	0.84 (3)	2.21 (3)	2.961 (4)	151 (2)

Symmetry codes: (i) 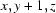; (ii) 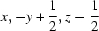; (iii) 
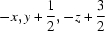
; (iv) 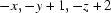; (v) 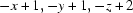.

**Fig. 1.**
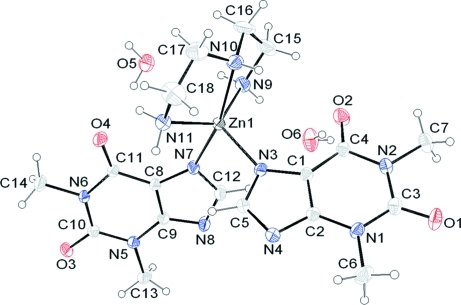
Molecular structure of the title compound with displacement ellipsoids at the 50% probability level.

## supplementary crystallographic information

### Comment

Research concerning transition metal complexes of theophylline have attracted considerable interest because they serve as model compounds for the study of the interaction between metal ions and oxopurine bases of nucleic acids. In basic media, the theophylline molecule acts as a monodentate anionic ligand and coordinates through the N(7) atom (Begum *et al.*,1994; Birdsall & Zitzman, 1979; Bombicz *et al.*, 1997). We report here the crystal structure of (diethylenetriamine)bis(theophyllinato)zinc(II) dihydrate containing deprotonated theophyllinato anion ligands. The diethylenetriamine acts as a tridentate chelating ligand. The crystal structure is built of discrete molecules of the Zn(II) complex (Fig. 1). The Zn(II) ion is five-coordinate in a distorted trigonal-bipyramidal environment. The terminal nitrogen atoms of diethylenetriamine and the N3 nitrogen atom of one theophyllinato ion define the equatorial plane. The second theophyllinato anion and the middle nitrogen atom of diethylenetriamine occupy the axial positions, with a N7—Zn1—N10 angle of 165.13 (12)°.

The isostructural copper(II) complex had been described earlier (Sorrell *et al., *1976).

### Experimental

To a suspension of theophylline (0.4 g, 2.22 mmol) in water (15 cm^3^), diethylenetriamine (0.5 cm^3^) was added. The obtained clear solution was mixed with a solution of Zn(CH_3_COO)_2 _.2 H_2_O (0.2195 g, 1 mmol) in a diethylenetriamine–water mixture (1 cm^3^ of diethylenetriamine in 5 cm^3^ of water). The reaction mixture was stirred at 50°C for 30 min. and stored at room temperature over night. The white polycrystalline powder formed was collected by filtration, washed with aqueous diethylenetriamine (5%) and dried. Colourless single crystals were obtained by recrystallization from aqueous solutions after standing at room temperature 6 days.

### Refinement

All hydrogen atoms except those in the two water molecules were placed in calculated positions using a riding model, with C—H = 0.93–0.97 Å and with *U*_iso_= 1.5*U*_eq_ (C) for methyl H and *U*_iso_= 1.2*U*_eq_ (C) for aryl H. The methyl groups were allowed to rotate but not to tip. Hydrogen atoms from the two water molecules were found from difference map and refined with a restrained O—H distance of 0.82 (2) Å, 0.825 (19), 0.82 (2)Å and 0.814 (19) Å, respectively.

### Crystal data

**Table:**

[Zn(C_7_H_7_N_4_O_2_)_2_(C_4_H_13_N_3_)]·2H_2_O	*F*(000) = 1176
*M**_r_* = 562.91	*D*_x_ = 1.558 Mg m^−^^3^
Monoclinic, *P*2_1_/*c*	Mo *K*α radiation, λ = 0.71073 Å
Hall symbol: -P 2ybc	Cell parameters from 3820 reflections
*a* = 18.4655 (13) Å	θ = 2.6–22.8°
*b* = 8.2603 (6) Å	µ = 1.08 mm^−^^1^
*c* = 15.9252 (12) Å	*T* = 297 K
β = 98.904 (1)°	Block, colourless
*V* = 2399.8 (3) Å^3^	0.26 × 0.25 × 0.15 mm
*Z* = 4	

### Data collection

**Table:**

Bruker SMART APEX diffractometer	4214 independent reflections
Radiation source: fine-focus sealed tube	3800 reflections with *I* > 2σ(*I*)
graphite	*R*_int_ = 0.041
φ and ω scans	θ_max_ = 25.0°, θ_min_ = 2.2°
Absorption correction: multi-scan (*SADABS*; Bruker, 2001)	*h* = −21→21
*T*_min_ = 0.766, *T*_max_ = 0.854	*k* = −9→9
16801 measured reflections	*l* = −18→18

### Refinement

**Table:**

Refinement on *F*^2^	Primary atom site location: structure-invariant direct methods
Least-squares matrix: full	Secondary atom site location: difference Fourier map
*R*[*F*^2^ > 2σ(*F*^2^)] = 0.049	Hydrogen site location: inferred from neighbouring sites
*wR*(*F*^2^) = 0.115	H atoms treated by a mixture of independent and constrained refinement
*S* = 1.15	*w* = 1/[σ^2^(*F*_o_^2^) + (0.0449*P*)^2^ + 2.691*P*] where *P* = (*F*_o_^2^ + 2*F*_c_^2^)/3
4214 reflections	(Δ/σ)_max_ = 0.001
349 parameters	Δρ_max_ = 0.53 e Å^−^^3^
5 restraints	Δρ_min_ = −0.34 e Å^−^^3^

### Special details

**Table:**

Geometry. All e.s.d.'s (except the e.s.d. in the dihedral angle between two l.s. planes) are estimated using the full covariance matrix. The cell e.s.d.'s are taken into account individually in the estimation of e.s.d.'s in distances, angles and torsion angles; correlations between e.s.d.'s in cell parameters are only used when they are defined by crystal symmetry. An approximate (isotropic) treatment of cell e.s.d.'s is used for estimating e.s.d.'s involving l.s. planes.
Refinement. Refinement of *F*^2^ against ALL reflections. The weighted *R*-factor *wR* and goodness of fit *S* are based on *F*^2^, conventional *R*-factors *R* are based on *F*, with *F* set to zero for negative *F*^2^. The threshold expression of *F*^2^ > σ(*F*^2^) is used only for calculating *R*-factors(gt) *etc*. and is not relevant to the choice of reflections for refinement. *R*-factors based on *F*^2^ are statistically about twice as large as those based on *F*, and *R*- factors based on ALL data will be even larger.

### Fractional atomic coordinates and isotropic or equivalent isotropic displacement parameters (Å^2^)

**Table:**

	*x*	*y*	*z*	*U*_iso_*/*U*_eq_	
H2	0.090 (3)	0.882 (7)	0.814 (4)	0.12 (3)*	
H1	0.111 (3)	1.030 (3)	0.801 (3)	0.064 (15)*	
H3	0.3221 (19)	0.383 (6)	0.733 (3)	0.077 (19)*	
H4	0.303 (3)	0.295 (5)	0.670 (3)	0.066 (16)*	
C1	0.36750 (17)	0.4373 (4)	0.9496 (2)	0.0274 (7)	
C2	0.38322 (18)	0.3506 (4)	1.0237 (2)	0.0285 (7)	
C3	0.4959 (2)	0.2550 (4)	0.9884 (2)	0.0354 (8)	
C4	0.41449 (18)	0.4342 (4)	0.8878 (2)	0.0315 (8)	
C5	0.28123 (19)	0.4589 (4)	1.0227 (2)	0.0339 (8)	
H5	0.2372	0.4892	1.0397	0.041*	
C6	0.4625 (3)	0.1727 (5)	1.1240 (3)	0.0562 (12)	
H6A	0.5146	0.1634	1.1406	0.084*	
H6B	0.4412	0.0666	1.1168	0.084*	
H6C	0.4419	0.2298	1.1672	0.084*	
C7	0.5323 (2)	0.3326 (5)	0.8547 (3)	0.0483 (10)	
H7A	0.5809	0.3389	0.8863	0.072*	
H7B	0.5247	0.4205	0.8149	0.072*	
H7C	0.5264	0.2316	0.8245	0.072*	
C8	0.06859 (17)	0.5122 (4)	0.8720 (2)	0.0276 (7)	
C9	0.04497 (18)	0.3547 (4)	0.8627 (2)	0.0287 (7)	
C10	−0.07285 (18)	0.4197 (4)	0.8940 (2)	0.0304 (8)	
C11	0.02094 (18)	0.6365 (4)	0.8909 (2)	0.0297 (8)	
C12	0.1516 (2)	0.3635 (4)	0.8352 (3)	0.0387 (9)	
H12	0.1959	0.3309	0.8197	0.046*	
C13	−0.0440 (2)	0.1359 (4)	0.8707 (3)	0.0462 (10)	
H13A	−0.0850	0.1176	0.8999	0.069*	
H13B	−0.0034	0.0704	0.8959	0.069*	
H13C	−0.0573	0.1074	0.8119	0.069*	
C14	−0.1046 (2)	0.7002 (5)	0.9130 (3)	0.0489 (11)	
H14A	−0.1453	0.6921	0.8677	0.073*	
H14B	−0.0837	0.8066	0.9131	0.073*	
H14C	−0.1212	0.6810	0.9663	0.073*	
C15	0.3002 (3)	0.7952 (6)	0.7322 (3)	0.0603 (13)	
H15A	0.2926	0.8333	0.6739	0.072*	
H15B	0.3446	0.7308	0.7412	0.072*	
C16	0.3077 (3)	0.9341 (6)	0.7908 (3)	0.0646 (14)	
H16A	0.2670	1.0080	0.7750	0.077*	
H16B	0.3527	0.9917	0.7862	0.077*	
C17	0.2874 (3)	1.0025 (6)	0.9349 (4)	0.0683 (14)	
H17A	0.2525	1.0755	0.9025	0.082*	
H17B	0.3300	1.0652	0.9588	0.082*	
C18	0.2545 (3)	0.9303 (7)	1.0032 (3)	0.0739 (15)	
H18A	0.2349	1.0150	1.0353	0.089*	
H18B	0.2918	0.8727	1.0414	0.089*	
N1	0.44707 (15)	0.2614 (4)	1.04420 (18)	0.0335 (7)	
N2	0.47870 (15)	0.3428 (4)	0.91318 (18)	0.0340 (7)	
N3	0.29968 (14)	0.5092 (3)	0.94963 (18)	0.0305 (6)	
N4	0.32938 (16)	0.3619 (3)	1.07095 (18)	0.0338 (7)	
N5	−0.02331 (15)	0.3060 (3)	0.87753 (19)	0.0318 (7)	
N6	−0.04898 (14)	0.5793 (3)	0.90045 (18)	0.0310 (6)	
N7	0.13966 (15)	0.5172 (3)	0.85451 (19)	0.0322 (7)	
N8	0.09705 (15)	0.2580 (4)	0.8390 (2)	0.0373 (7)	
N9	0.23797 (17)	0.6963 (4)	0.74736 (19)	0.0414 (8)	
H9A	0.2419	0.5973	0.7249	0.050*	
H9B	0.1963	0.7415	0.7208	0.050*	
N10	0.30890 (18)	0.8803 (4)	0.8791 (2)	0.0423 (8)	
N11	0.19553 (17)	0.8177 (4)	0.9694 (2)	0.0401 (7)	
H11A	0.1554	0.8734	0.9467	0.048*	
H11B	0.1838	0.7552	1.0116	0.048*	
O1	0.55292 (14)	0.1781 (3)	1.00333 (18)	0.0511 (7)	
O2	0.40477 (14)	0.4991 (4)	0.81732 (16)	0.0483 (7)	
O3	−0.13559 (13)	0.3833 (3)	0.90348 (17)	0.0390 (6)	
O4	0.03442 (14)	0.7823 (3)	0.89879 (19)	0.0457 (7)	
O5	0.11729 (18)	0.9342 (4)	0.7883 (2)	0.0525 (7)	
O6	0.2845 (2)	0.3503 (5)	0.7033 (2)	0.0656 (9)	
Zn1	0.23190 (2)	0.67315 (5)	0.87641 (2)	0.02884 (14)	
H10	0.3487 (14)	0.837 (4)	0.899 (2)	0.034 (11)*	

### Atomic displacement parameters (Å^2^)

**Table:**

	*U*^11^	*U*^22^	*U*^33^	*U*^12^	*U*^13^	*U*^23^
C1	0.0239 (17)	0.0277 (17)	0.0301 (18)	−0.0005 (14)	0.0028 (14)	−0.0007 (14)
C2	0.0288 (18)	0.0260 (17)	0.0304 (18)	−0.0010 (14)	0.0036 (14)	−0.0025 (14)
C3	0.0291 (19)	0.039 (2)	0.036 (2)	0.0001 (16)	−0.0010 (15)	−0.0070 (16)
C4	0.0286 (18)	0.0322 (19)	0.0332 (19)	−0.0001 (15)	0.0026 (15)	−0.0018 (15)
C5	0.0290 (19)	0.0335 (19)	0.041 (2)	0.0038 (15)	0.0124 (16)	0.0039 (16)
C6	0.058 (3)	0.063 (3)	0.046 (2)	0.022 (2)	0.001 (2)	0.020 (2)
C7	0.038 (2)	0.067 (3)	0.043 (2)	0.004 (2)	0.0171 (18)	−0.006 (2)
C8	0.0238 (17)	0.0244 (17)	0.0352 (19)	0.0013 (13)	0.0062 (14)	0.0029 (14)
C9	0.0250 (17)	0.0251 (17)	0.0364 (19)	−0.0020 (14)	0.0055 (14)	−0.0019 (14)
C10	0.0270 (18)	0.0306 (18)	0.0340 (19)	0.0015 (15)	0.0063 (15)	0.0035 (15)
C11	0.0251 (18)	0.0255 (18)	0.0388 (19)	0.0033 (14)	0.0056 (15)	0.0042 (14)
C12	0.0279 (19)	0.031 (2)	0.058 (2)	−0.0012 (15)	0.0122 (17)	−0.0094 (17)
C13	0.041 (2)	0.0244 (19)	0.076 (3)	−0.0059 (16)	0.016 (2)	−0.0028 (19)
C14	0.034 (2)	0.034 (2)	0.082 (3)	0.0075 (17)	0.020 (2)	−0.001 (2)
C15	0.055 (3)	0.079 (3)	0.051 (3)	−0.008 (2)	0.018 (2)	0.025 (2)
C16	0.055 (3)	0.061 (3)	0.079 (3)	−0.014 (2)	0.012 (2)	0.037 (3)
C17	0.069 (3)	0.039 (2)	0.098 (4)	−0.022 (2)	0.017 (3)	−0.013 (3)
C18	0.087 (4)	0.070 (3)	0.066 (3)	−0.027 (3)	0.019 (3)	−0.030 (3)
N1	0.0323 (16)	0.0346 (16)	0.0322 (16)	0.0086 (13)	0.0007 (13)	0.0038 (13)
N2	0.0273 (15)	0.0428 (18)	0.0329 (16)	0.0022 (13)	0.0073 (12)	−0.0020 (13)
N3	0.0238 (14)	0.0297 (15)	0.0376 (16)	0.0020 (12)	0.0031 (12)	0.0070 (13)
N4	0.0340 (16)	0.0342 (16)	0.0346 (16)	0.0023 (13)	0.0097 (13)	0.0046 (13)
N5	0.0281 (15)	0.0236 (14)	0.0450 (17)	−0.0029 (12)	0.0095 (13)	0.0013 (12)
N6	0.0219 (14)	0.0273 (15)	0.0456 (17)	0.0038 (12)	0.0111 (13)	0.0034 (13)
N7	0.0239 (15)	0.0273 (15)	0.0469 (18)	−0.0017 (12)	0.0103 (13)	−0.0022 (13)
N8	0.0282 (16)	0.0282 (16)	0.057 (2)	−0.0001 (13)	0.0113 (14)	−0.0079 (14)
N9	0.0367 (17)	0.0494 (19)	0.0383 (18)	0.0024 (15)	0.0058 (14)	0.0081 (15)
N10	0.0326 (18)	0.0337 (17)	0.059 (2)	−0.0045 (15)	0.0026 (16)	0.0033 (16)
N11	0.0415 (18)	0.0365 (17)	0.0432 (18)	0.0012 (14)	0.0092 (14)	−0.0008 (14)
O1	0.0320 (15)	0.0621 (19)	0.0577 (18)	0.0190 (14)	0.0019 (13)	−0.0007 (14)
O2	0.0422 (16)	0.0691 (19)	0.0353 (15)	0.0063 (14)	0.0111 (12)	0.0141 (14)
O3	0.0256 (13)	0.0393 (14)	0.0547 (16)	−0.0036 (11)	0.0146 (11)	0.0010 (12)
O4	0.0357 (14)	0.0243 (13)	0.080 (2)	−0.0025 (11)	0.0175 (14)	−0.0025 (13)
O5	0.062 (2)	0.0422 (18)	0.0586 (19)	0.0043 (16)	0.0245 (16)	−0.0083 (15)
O6	0.054 (2)	0.081 (3)	0.063 (2)	0.0078 (19)	0.0140 (18)	−0.0266 (19)
Zn1	0.0249 (2)	0.0272 (2)	0.0351 (2)	−0.00167 (16)	0.00696 (16)	0.00309 (17)

### Geometric parameters (Å, °)

**Table:**

C1—C2	1.372 (5)	C13—H13A	0.9600
C1—N3	1.386 (4)	C13—H13B	0.9600
C1—C4	1.410 (5)	C13—H13C	0.9600
C2—N4	1.339 (4)	C14—N6	1.468 (4)
C2—N1	1.386 (4)	C14—H14A	0.9600
C3—O1	1.221 (4)	C14—H14B	0.9600
C3—N1	1.362 (5)	C14—H14C	0.9600
C3—N2	1.393 (5)	C15—N9	1.459 (5)
C4—O2	1.231 (4)	C15—C16	1.472 (7)
C4—N2	1.411 (4)	C15—H15A	0.9700
C5—N3	1.329 (4)	C15—H15B	0.9700
C5—N4	1.346 (4)	C16—N10	1.472 (6)
C5—H5	0.9300	C16—H16A	0.9700
C6—N1	1.456 (5)	C16—H16B	0.9700
C6—H6A	0.9600	C17—N10	1.441 (6)
C6—H6B	0.9600	C17—C18	1.454 (7)
C6—H6C	0.9600	C17—H17A	0.9700
C7—N2	1.463 (4)	C17—H17B	0.9700
C7—H7A	0.9600	C18—N11	1.469 (5)
C7—H7B	0.9600	C18—H18A	0.9700
C7—H7C	0.9600	C18—H18B	0.9700
C8—C9	1.373 (5)	N3—Zn1	2.076 (3)
C8—N7	1.384 (4)	N7—Zn1	2.121 (3)
C8—C11	1.415 (4)	N9—Zn1	2.084 (3)
C9—N8	1.348 (4)	N9—H9A	0.9000
C9—N5	1.378 (4)	N9—H9B	0.9000
C10—O3	1.229 (4)	N10—Zn1	2.221 (3)
C10—N5	1.365 (4)	N10—H10	0.834 (19)
C10—N6	1.388 (4)	N11—Zn1	2.093 (3)
C11—O4	1.232 (4)	N11—H11A	0.9000
C11—N6	1.405 (4)	N11—H11B	0.9000
C12—N7	1.333 (4)	O5—H2	0.82 (6)
C12—N8	1.341 (5)	O5—H1	0.825 (19)
C12—H12	0.9300	O6—H3	0.82 (2)
C13—N5	1.456 (4)	O6—H4	0.81 (5)
			
C2—C1—N3	107.1 (3)	N10—C16—H16B	109.5
C2—C1—C4	121.4 (3)	H16A—C16—H16B	108.1
N3—C1—C4	131.4 (3)	N10—C17—C18	111.2 (4)
N4—C2—C1	111.7 (3)	N10—C17—H17A	109.4
N4—C2—N1	125.7 (3)	C18—C17—H17A	109.4
C1—C2—N1	122.6 (3)	N10—C17—H17B	109.4
O1—C3—N1	122.0 (3)	C18—C17—H17B	109.4
O1—C3—N2	120.9 (3)	H17A—C17—H17B	108.0
N1—C3—N2	117.0 (3)	C17—C18—N11	111.0 (4)
O2—C4—C1	127.6 (3)	C17—C18—H18A	109.4
O2—C4—N2	119.5 (3)	N11—C18—H18A	109.4
C1—C4—N2	112.9 (3)	C17—C18—H18B	109.4
N3—C5—N4	116.7 (3)	N11—C18—H18B	109.4
N3—C5—H5	121.6	H18A—C18—H18B	108.0
N4—C5—H5	121.6	C3—N1—C2	119.5 (3)
N1—C6—H6A	109.5	C3—N1—C6	119.4 (3)
N1—C6—H6B	109.5	C2—N1—C6	121.1 (3)
H6A—C6—H6B	109.5	C3—N2—C4	126.5 (3)
N1—C6—H6C	109.5	C3—N2—C7	115.5 (3)
H6A—C6—H6C	109.5	C4—N2—C7	118.0 (3)
H6B—C6—H6C	109.5	C5—N3—C1	102.7 (3)
N2—C7—H7A	109.5	C5—N3—Zn1	118.8 (2)
N2—C7—H7B	109.5	C1—N3—Zn1	138.2 (2)
H7A—C7—H7B	109.5	C2—N4—C5	101.7 (3)
N2—C7—H7C	109.5	C10—N5—C9	119.3 (3)
H7A—C7—H7C	109.5	C10—N5—C13	120.2 (3)
H7B—C7—H7C	109.5	C9—N5—C13	120.4 (3)
C9—C8—N7	107.6 (3)	C10—N6—C11	126.7 (3)
C9—C8—C11	120.9 (3)	C10—N6—C14	115.8 (3)
N7—C8—C11	131.4 (3)	C11—N6—C14	117.4 (3)
N8—C9—C8	111.3 (3)	C12—N7—C8	102.4 (3)
N8—C9—N5	125.9 (3)	C12—N7—Zn1	117.3 (2)
C8—C9—N5	122.8 (3)	C8—N7—Zn1	138.4 (2)
O3—C10—N5	121.9 (3)	C12—N8—C9	101.6 (3)
O3—C10—N6	121.2 (3)	C15—N9—Zn1	112.2 (3)
N5—C10—N6	117.0 (3)	C15—N9—H9A	109.2
O4—C11—N6	119.5 (3)	Zn1—N9—H9A	109.2
O4—C11—C8	127.5 (3)	C15—N9—H9B	109.2
N6—C11—C8	113.0 (3)	Zn1—N9—H9B	109.2
N7—C12—N8	117.1 (3)	H9A—N9—H9B	107.9
N7—C12—H12	121.4	C17—N10—C16	114.4 (4)
N8—C12—H12	121.4	C17—N10—Zn1	108.4 (3)
N5—C13—H13A	109.5	C16—N10—Zn1	107.4 (3)
N5—C13—H13B	109.5	C17—N10—H10	112 (3)
H13A—C13—H13B	109.5	C16—N10—H10	112 (3)
N5—C13—H13C	109.5	Zn1—N10—H10	102 (3)
H13A—C13—H13C	109.5	C18—N11—Zn1	108.6 (3)
H13B—C13—H13C	109.5	C18—N11—H11A	110.0
N6—C14—H14A	109.5	Zn1—N11—H11A	110.0
N6—C14—H14B	109.5	C18—N11—H11B	110.0
H14A—C14—H14B	109.5	Zn1—N11—H11B	110.0
N6—C14—H14C	109.5	H11A—N11—H11B	108.3
H14A—C14—H14C	109.5	H2—O5—H1	105 (6)
H14B—C14—H14C	109.5	H3—O6—H4	98 (5)
N9—C15—C16	109.3 (4)	N3—Zn1—N9	119.60 (12)
N9—C15—H15A	109.8	N3—Zn1—N11	101.91 (12)
C16—C15—H15A	109.8	N9—Zn1—N11	135.87 (13)
N9—C15—H15B	109.8	N3—Zn1—N7	95.46 (11)
C16—C15—H15B	109.8	N9—Zn1—N7	93.33 (12)
H15A—C15—H15B	108.3	N11—Zn1—N7	97.20 (12)
C15—C16—N10	110.9 (4)	N3—Zn1—N10	99.41 (12)
C15—C16—H16A	109.5	N9—Zn1—N10	79.40 (13)
N10—C16—H16A	109.5	N11—Zn1—N10	79.72 (13)
C15—C16—H16B	109.5	N7—Zn1—N10	165.13 (12)
			
N3—C1—C2—N4	0.4 (4)	O3—C10—N6—C14	−4.2 (5)
C4—C1—C2—N4	−175.9 (3)	N5—C10—N6—C14	176.4 (3)
N3—C1—C2—N1	179.3 (3)	O4—C11—N6—C10	−179.7 (3)
C4—C1—C2—N1	3.0 (5)	C8—C11—N6—C10	0.7 (5)
C2—C1—C4—O2	175.9 (4)	O4—C11—N6—C14	4.6 (5)
N3—C1—C4—O2	0.6 (6)	C8—C11—N6—C14	−174.9 (3)
C2—C1—C4—N2	−3.4 (5)	N8—C12—N7—C8	−0.8 (4)
N3—C1—C4—N2	−178.7 (3)	N8—C12—N7—Zn1	166.3 (3)
N7—C8—C9—N8	−0.8 (4)	C9—C8—N7—C12	0.9 (4)
C11—C8—C9—N8	175.6 (3)	C11—C8—N7—C12	−174.9 (4)
N7—C8—C9—N5	178.4 (3)	C9—C8—N7—Zn1	−161.7 (3)
C11—C8—C9—N5	−5.3 (5)	C11—C8—N7—Zn1	22.4 (6)
C9—C8—C11—O4	−178.1 (4)	N7—C12—N8—C9	0.4 (4)
N7—C8—C11—O4	−2.7 (6)	C8—C9—N8—C12	0.3 (4)
C9—C8—C11—N6	1.5 (5)	N5—C9—N8—C12	−178.8 (3)
N7—C8—C11—N6	176.9 (3)	C16—C15—N9—Zn1	−40.5 (4)
N9—C15—C16—N10	51.5 (5)	C18—C17—N10—C16	151.2 (4)
N10—C17—C18—N11	−51.8 (6)	C18—C17—N10—Zn1	31.4 (5)
O1—C3—N1—C2	−179.9 (3)	C15—C16—N10—C17	−156.8 (4)
N2—C3—N1—C2	1.0 (5)	C15—C16—N10—Zn1	−36.5 (4)
O1—C3—N1—C6	−0.6 (5)	C17—C18—N11—Zn1	44.9 (5)
N2—C3—N1—C6	−179.7 (3)	C5—N3—Zn1—N9	−159.7 (2)
N4—C2—N1—C3	177.2 (3)	C1—N3—Zn1—N9	27.1 (4)
C1—C2—N1—C3	−1.6 (5)	C5—N3—Zn1—N11	35.9 (3)
N4—C2—N1—C6	−2.2 (5)	C1—N3—Zn1—N11	−137.4 (3)
C1—C2—N1—C6	179.1 (3)	C5—N3—Zn1—N7	−62.7 (3)
O1—C3—N2—C4	179.0 (3)	C1—N3—Zn1—N7	124.1 (3)
N1—C3—N2—C4	−2.0 (5)	C5—N3—Zn1—N10	117.2 (3)
O1—C3—N2—C7	1.1 (5)	C1—N3—Zn1—N10	−56.0 (4)
N1—C3—N2—C7	−179.8 (3)	C15—N9—Zn1—N3	−79.2 (3)
O2—C4—N2—C3	−176.3 (3)	C15—N9—Zn1—N11	78.7 (3)
C1—C4—N2—C3	3.1 (5)	C15—N9—Zn1—N7	−177.4 (3)
O2—C4—N2—C7	1.5 (5)	C15—N9—Zn1—N10	15.6 (3)
C1—C4—N2—C7	−179.1 (3)	C18—N11—Zn1—N3	77.1 (3)
N4—C5—N3—C1	0.1 (4)	C18—N11—Zn1—N9	−83.3 (3)
N4—C5—N3—Zn1	−175.3 (2)	C18—N11—Zn1—N7	174.3 (3)
C2—C1—N3—C5	−0.2 (4)	C18—N11—Zn1—N10	−20.4 (3)
C4—C1—N3—C5	175.5 (4)	C12—N7—Zn1—N3	−43.4 (3)
C2—C1—N3—Zn1	173.7 (3)	C8—N7—Zn1—N3	117.4 (3)
C4—C1—N3—Zn1	−10.5 (6)	C12—N7—Zn1—N9	76.8 (3)
C1—C2—N4—C5	−0.3 (4)	C8—N7—Zn1—N9	−122.4 (4)
N1—C2—N4—C5	−179.2 (3)	C12—N7—Zn1—N11	−146.2 (3)
N3—C5—N4—C2	0.1 (4)	C8—N7—Zn1—N11	14.6 (4)
O3—C10—N5—C9	176.3 (3)	C12—N7—Zn1—N10	136.8 (5)
N6—C10—N5—C9	−4.2 (5)	C8—N7—Zn1—N10	−62.4 (7)
O3—C10—N5—C13	0.0 (5)	C17—N10—Zn1—N3	−106.1 (3)
N6—C10—N5—C13	179.4 (3)	C16—N10—Zn1—N3	129.9 (3)
N8—C9—N5—C10	−174.3 (3)	C17—N10—Zn1—N9	135.3 (3)
C8—C9—N5—C10	6.7 (5)	C16—N10—Zn1—N9	11.3 (3)
N8—C9—N5—C13	2.1 (5)	C17—N10—Zn1—N11	−5.6 (3)
C8—C9—N5—C13	−176.9 (3)	C16—N10—Zn1—N11	−129.6 (3)
O3—C10—N6—C11	−179.9 (3)	C17—N10—Zn1—N7	73.7 (6)
N5—C10—N6—C11	0.7 (5)	C16—N10—Zn1—N7	−50.4 (6)

### Hydrogen-bond geometry (Å, °)

**Table:**

*D*—H···*A*	*D*—H	H···*A*	*D*···*A*	*D*—H···*A*
O5—H1···N8^i^	0.83 (2)	2.01 (3)	2.835 (5)	177 (3)
O5—H2···O4	0.82 (6)	2.00 (6)	2.801 (5)	167 (6)
O6—H3···O2	0.82 (2)	2.10 (4)	2.914 (4)	169 (4)
O6—H4···N4^ii^	0.81 (5)	2.16 (6)	2.957 (5)	168 (4)
N9—H9A···O6	0.90	2.23	3.096 (5)	161
N9—H9B···O3^iii^	0.90	2.42	3.213 (4)	147 (2)
N11—H11B···O3^iv^	0.90	2.07	2.962 (4)	169 (2)
N10—H10···O1^v^	0.84 (3)	2.21 (3)	2.961 (4)	151 (2)

Symmetry codes: (i) *x*, *y*+1, *z*; (ii) *x*, −*y*+1/2, *z*−1/2; (iii) −*x*, *y*+1/2, −*z*+3/2; (iv) −*x*, −*y*+1, −*z*+2; (v) −*x*+1, −*y*+1, −*z*+2.
